# Strategic Approaches for Colon Targeted Drug Delivery: An Overview of Recent Advancements

**DOI:** 10.3390/pharmaceutics12010068

**Published:** 2020-01-15

**Authors:** Sang Hoon Lee, Rajiv Bajracharya, Jeong Youn Min, Ji-Won Han, Byeong Ju Park, Hyo-Kyung Han

**Affiliations:** College of Pharmacy, Dongguk University-Seoul, Dongguk-ro 32, Ilsan-Donggu, Goyang 10326, Korea; sh_lee@dongguk.edu (S.H.L.); rajivbajra@hotmail.com (R.B.); jyin14@naver.com (J.Y.M.); hjw0868@naver.com (J.-W.H.); bjpark@dongguk.edu (B.J.P.)

**Keywords:** colon, noninvasive drug delivery, inflammatory bowel diseases, colorectal cancer, protein drugs

## Abstract

Colon targeted drug delivery systems have gained a great deal of attention as potential carriers for the local treatment of colonic diseases with reduced systemic side effects and also for the enhanced oral delivery of various therapeutics vulnerable to acidic and enzymatic degradation in the upper gastrointestinal tract. In recent years, the global pharmaceutical market for biologics has grown, and increasing demand for a more patient-friendly drug administration system highlights the importance of colonic drug delivery as a noninvasive delivery approach for macromolecules. Colon-targeted drug delivery systems for macromolecules can provide therapeutic benefits including better patient compliance (because they are pain-free and can be self-administered) and lower costs. Therefore, to achieve more efficient colonic drug delivery for local or systemic drug effects, various strategies have been explored including pH-dependent systems, enzyme-triggered systems, receptor-mediated systems, and magnetically-driven systems. In this review, recent advancements in various approaches for designing colon targeted drug delivery systems and their pharmaceutical applications are covered with a particular emphasis on formulation technologies.

## 1. Introduction

In the past few decades, the prevalence of colonic diseases has increased worldwide, demanding the effective local treatment of colonic diseases for more efficacious and safer drug therapies. Among colonic diseases, colorectal cancer (CRC) causes the most cancer-related deaths in Europe (accounting more than 200,000 deaths annually) [1], and it is the third most commonly diagnosed cancer worldwide [1,2]. The incidence of inflammatory bowel disease (IBD) is also increasing at an alarming rate in previously low-incidence areas such as Asia [3]. Consequently, the effective treatment of colonic diseases has become an important worldwide public healthcare issue.

For the local treatment of colonic diseases, colon-targeted drug delivery systems have been actively pursued since conventional non-targeted therapy may have undesirable side-effects and low efficacy due to the systemic absorption of drug before reaching the target site [4,5]. In addition to the topical delivery, colon-targeted drug delivery systems are also applicable to improve the bioavailability of drugs vulnerable to acidic and/or enzymatic destabilization in the upper gastrointestinal (GI) tract, particularly macromolecules such as proteins and peptides due to lower protease activity in the colon [6,7,8]. Although colonic delivery of macromolecules has been explored less extensively compared to small molecules, continuous research may reveal its potential as an effective oral delivery system for macromolecules.

Colon targeted drug delivery systems are designed to selectively release a drug in response to the colonic environment without premature drug release in the upper GI tract. Therefore, it is imperative to consider the physiological properties of the colon and the microenvironment surrounding disease site(s) for the successful development of colon-targeted drug delivery systems. In general, GI tract undergoes dynamic changes in motility, fluid contents, enzymatic activity, and pH from the stomach to the intestine [9]. Furthermore, the microenvironment surrounding a disease site in the colon is markedly different from normal and healthy regions. Patients with colonic diseases produce high levels of reactive oxygen species (ROS) and inflammatory cytokines, have an imbalance of important antioxidants, and suffer from mucosal injury [10]. Given that the pathophysiological changes in the microenvironment surrounding disease sites should be considered during formulation development, various formulation approaches have been explored to optimize the colonic drug delivery, including pH-sensitive systems, enzyme-triggered systems, and magnetically-driven systems. To enhance the specificity at disease sites, receptor-mediated systems have also been studied, which preferentially interact with specific receptors overexpressed at the site(s) of the disease. This review covers recent advancements in various formulation approaches in designing colon-targeted drug delivery systems and their pharmaceutical applications.

## 2. Formulation Approaches for Colon Targeted Drug Delivery

### 2.1. pH-Dependent Drug Delivery Systems

The colon exhibits a relatively higher pH than the upper GI tract, and this can be used as a targeting strategy for colonic drug delivery. Accordingly, a colon-targeted drug delivery system is designed by using pH-dependent polymers such as cellulose acetate phthalates (CAP), hydroxypropyl methyl-cellulose phthalate (HPMCP) 50 and 55, copolymers of methacrylic acid and methyl methacrylate (e.g., Eudragit^®^ S 100, Eudragit^®^ L, Eudragit^®^ FS, and Eudragit^®^ P4135 F) [11,12]. Particularly, Eudragit^®^ polymers are the most widely used synthetic copolymers for colonic drug delivery that offer mucoadhesiveness and pH-dependent drug release [13,14]. The ideal polymer should be able to withstand the low pH of the stomach and the proximal part of the small intestine but be dissolved by the pH of the terminal ileum and the colon. As a result, drug delivery systems coated with pH-dependent polymers having a dissolution threshold of pH 6.0–7.0 are expected to delay the drug dissolution and prevent premature drug release in the upper GI tract before reaching colonic sites [15]. However, this pH-dependent system has demonstrated significant variability in drug release and failure in vivo due to the vast inter- and intra-subject variability in critical parameters including pH, fluids volumes, GI transit times, and motility [16]. Furthermore, pH ranges of GI tract can be significantly altered by diet, disease state, water intake, and microbial metabolism [17]. For example, patients with ulcerative colitis exhibit more acidic colonic pH compared to healthy humans, leading to incomplete drug release from enteric coated systems at the target site [16]. Thus, the dynamic pH change by many internal and external factors may attenuate the efficiency of pH-dependent drug release systems, often leading to poorly site-selective drug release. Ibekwe et al. [18] also revealed that Eudragit^®^ S coating was not suitable for the colon-targeted drug release, either due to disintegration failure at the target site or early drug release before the target site. In the subsequent human studies, Ibekwe et al. [19] confirmed the lack of site-selective drug release of Eudragit^®^ S coated tablets, suggesting that disintegration of these tablets is affected by multiple physiological factors including gastrointestinal pH, feed status, and intestinal transit time.

To overcome this limitation of pH-dependent delivery systems, there have been attempts to use the combination of pH-dependent systems with other delivery systems including time-dependent systems and enzyme-triggered systems. For example, Eudragit^®^ S were blended with high-amylose maize starch for the integration of pH-dependent system and colonic microbial degradation systems [16,20]. Liu et al. [21] adopted dual coating approach by using the alkaline aqueous solution of Eudragit^®^ S with buffering agents for inner layer and the organic solution of Eudragit^®^ S for outer layer, accelerating the drug dissolution at pH > 7. Subsequently, Varum et al. [22] evaluated in vivo performance of this dual coated system in humans, demonstrating more consistent disintegration of dual coated tablets mainly in the lower intestinal tract. Hashem et al. [23] developed microspheres combining time-and pH-dependent systems for colonic delivery of prednisolone. By using a combination of Eudragit^®^ S and ethyl cellulose, they achieved greater colonic drug delivery while preventing premature drug release in the upper intestine [23]. Eudracol^®^ is another example of a multi-unit technology providing targeted drug delivery to the colon, with delayed and uniform drug release. This system is based on coating the pellet with Eudragit^®^ RL/RS and Eudragit^®^ FS 30D, providing colon-specific drug release in a pH-and time-dependent manner [24]. Overall, integrated systems of the different release-triggering mechanisms are more helpful to overcome the pathophysiological variability compared to pH-dependent system alone, although there is still need for further improvement. In addition, nano-/micro-particles also hold great potential for specifically targeting inflamed colonic tissues and enhance drug uptake. Accordingly, various formulations that have combined a pH-dependent system with particle size reduction have been developed for colon-targeted drug delivery.

#### 2.1.1. Polymer-Based Nano-/Micro-Particles

Many studies have demonstrated that pH-dependent polymeric nanoparticles are effective as colonic drug delivery systems [25,26]. Mutalik et al. [27] used novel pH-sensitive hydrolyzed polyacrylamide-grafted-xanthan gum (PAAm-g-XG) for the colon-targeted delivery of curcumin nanoparticles. The amount of drug released from the PAAm-g-XG-modified nanoparticles was minimal in acidic conditions (pH 1.2 and 4.5), while faster and higher drug release from nanoparticles was observed at pH 7.2 [27]. Accordingly, the nanoparticles were effective in attenuating colonic inflammation and weight loss in IBD rat models. Furthermore, the blended mixture of two different pH-sensitive polymers can be used to control the drug release rate. Sahu and Pandey [28] developed the HBsAg-loaded nanoparticles by using the combination of Eudragit^®^ L100 and Eudragit^®^ S100 for effective colonic immunization, confirming the effective distribution of nanoparticles at the colon along with the improved immune response [28]. To improve the site-specificity to the colon, Naeem et al. [29] fabricated budesonide-loaded pH-/time-dependent nanoparticles for the effective treatment of colitis. These nanoparticles were prepared with Eudragit^®^ FS30D and Eudragit^®^ RS100, using an oil-in-water emulsion solvent evaporation method. Eudragit^®^ FS30D is a pH-dependent polymer that dissolves in an environment above pH 7.0, while Eudragit^®^ RS100 is a time-dependent, controlled-release polymer having low permeability. Combining these two polymers effectively minimized premature drug release in the upper GI tract and achieved sustained-drug release throughout the colon. Furthermore, in colitis mice models, these pH-/time-dependent nanoparticles delivered drugs more efficiently to the inflamed colonic sites [29].

#### 2.1.2. Lipid-Based Formulations

Liposomes are an efficient drug delivery system composed of double-layered phospholipids [10,30,31]. Liposomes are biodegradable, biocompatible, and amenable to the incorporation of both hydrophilic and lipophilic drugs [32,33]. The surface of liposomes can be coated with pH-dependent polymers to avoid the destabilization of liposomes in acidic conditions and also with ligands to improve the site-specificity. For example, Zhao et al. [34] developed colon-targeted liposomal formulations for sorafenib by coating the surface of anionic liposomes with glycol chitosan and pH-dependent Eudragit^®^ S100. These liposomes showed high stability at acidic and neutral pHs with minimal drug leakage, which enhanced the systemic exposure of sorafenib in rats [34].

Solid lipid nanoparticles are also a superior system in terms of drug protection, entrapment efficiency, and increasing the amount of drug released at specific sites [10,35,36]. The lipid matrix of solid lipid nanoparticles degrades at a slow rate and allows for extended drug release [10].

Self-microemulsifying drug delivery system (SMEDDS) have immense potential for enhancing the oral bioavailability of various hydrophobic drugs, which can be useful in the design of colon-targeted drug delivery systems [37,38,39,40,41]. Zhang et al. [42] prepared folate-modified SMEDDS (FSMEDDS) containing curcumin, which were then filled into soft capsules coated with Eudragit^®^ S 100. This curcumin-loaded FSMEDDS formulation efficiently bound to folate receptors on colon cancer cells. These results demonstrated that colon-targeted FSMEDDS capsules are a viable means through which curcumin can be delivered to the colon [42].

#### 2.1.3. Tablets and Capsules

**Figure 1:**
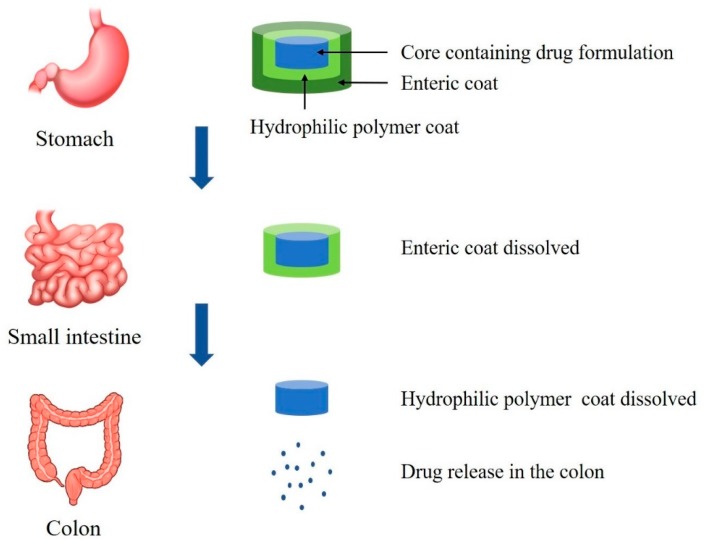
Drug release in the colon from pH-sensitive polymer-based system.

Colon targeted drug delivery can be achieved with film coated tablets or capsules [4,43] even though there are few commercially available products. Figure 1 is a schematic diagram that illustrates colonic drug release from a pH-sensitive polymer-coated drug delivery system. This system is applicable to macromolecules as well as low molecular synthetic drugs. Recently, Crowe et al. [44] developed the Eudragit L100-coated tablets for the colonic delivery of a novel anti-tumor necrosis factor α domain antibody (V565). This tablet exhibited the sustained drug release at pH ≥ 6 but no drug release during 2-hr incubation in acidic conditions. In vivo studies in monkeys also supported the sustained release of V565 in the intestine for the topical treatment of IBD [44]. In addition, the drug release profiles can be manipulated by using a combination of copolymers with varying the ratios [44]. This combination system may be superior to tablets coated with a single polymer for colon-targeted drug delivery. However, the tablets coated only with pH-sensitive enteric polymers still face the issues of premature drug release due to the variability of pH in GI tract [45]. In addition, variability in the GI fluid composition, feeding status, and GI transit time affect the site-specific drug release from the pH-dependent system [45]. Therefore, there have been continuous efforts to improve the targeting effectiveness via the multi-unit formulations based on the integration of the different mechanism-based systems with pH-dependent coating [46]. For example, Park et al. [46] prepared a bisacodyl-loaded multi-unit tablet by coating with different combinations of pH-dependent polymers (Eudragit S and Eudragit L) and time-dependent polymer (Eudragit RS). Drug release from the optimized tablet was minimal in the simulated gastric and intestinal fluids while extensive drug release was observed in the colonic fluid [46]. Recently, Foppoli et al. [47] also reported the effective colonic delivery system of 5-aminosalicylic acid based on the combination of time-dependent and pH-dependent approaches, which was prepared by successive coating of a tablet core with low-viscosity HPMC and Eudragit^®^ L. Furthermore, based on a γ-scintigraphy study in human, they confirmed that there was no premature drug release before reaching the colon in both fed and fasted states [47].

Zein is a potential carrier for controlled-release solid dispersion systems delivering poorly water soluble drugs to the colon since it is resistant to low pH environments [48]. Recently, a single-layer film coating of tablets using biopolymer Zein in combination with Kollicoat^®^ MAE 100P showed high potential to prevent the drug release in the upper GI tract for the *delayed drug release* in the colon [49]. The ratio of the coating components and the thickness of the coating layer play an important role in the performance of coated tablets for colonic drug delivery.

In recent years, new coating technology has been actively pursued to improve the targeting effectiveness of pH-dependent delivery systems. For example, ColoPulse technology is an innovative pH responsive coating technology, which incorporates super-disintegrant in the coating matrix to accelerate the disintegration at the target site [50,51,52]. The incorporation of a super-disintegrant in a non-percolating mode leads to a more reliable and pulsatile drug release. Previous studies demonstrated that ColoPulse tablets enabled the site-specific delivery of the active substance to the ileo-colonic region of Crohn’s patients as well as healthy subjects [50,51]. Furthermore, food and time of food intake did not affect the targeting effectiveness of ColoPulse delivery systems [51]. Recently, Gareb et al. [52] adopted this technology to develop the ileo-colonic-targeted zero-order sustained-release tablets of budesonide for the topical treatment of IBD. The results indicated that drug release from the developed tablet began in the simulated ileum, and the release rate remained constant throughout the entire simulated colon [52]. They also developed and validated the production process of oral infliximab tablet coated with ColoPulse technology for the local treatment of ileo-colonic IBD [53]. Preparation of capsule shell with built-in gastroresistance is another approach for site-specific drug delivery. These gastroresistant capsule shells may have some advantages including large production using a typical high-speed capsule filler, encapsulation of diverse drugs, and potentially reducing research and development costs. Barbosa et al. [54] reported a simple method for producing enteric capsule shells without any additional coating steps. They prepared different enteric capsule shells to target various region of GI tract, by using cellulose derivatives (HPMC AS-LF and HP-55) along with acrylic/methacrylic acid derivatives (Eudragit^®^ L100 and Eudragit^®^ S100). Although the effectiveness of ready-made enteric capsules for colonic drug delivery has not been thoroughly evaluated yet, this may provide another option for targeted drug delivery.

### 2.2. Enzyme-Sensitive Drug Delivery Systems

#### 2.2.1. Polysaccharide-Based Systems

Microbiota-activated delivery systems have shown promise in colon-targeted drug delivery due to the abrupt increase of microbiota and the associated enzymatic activities in the lower GI tract. These systems are dependent on the specific enzyme activity of the colonic bacteria and the polymers degradable by colonic microorganisms. Particularly, polysaccharides such as pectin, guar gum, inulin, and chitosan have been used in colon-targeted drug delivery systems, because they can retain their integrity in the upper GI tract but are metabolized by colonic microflora to release the entrapped drug [55]. Recently, new polysaccharides including arabinoxylans and agave fructans are also being explored for colonic drug delivery systems [56,57]. Furthermore, structural modifications or derivatives of polysaccharides can improve drug release behavior, stability, and site specificity [58]. Mucoadhesiveness of polysaccharides can be advantageous for drug uptake via the prolonged contact between the mucosal surface and drug delivery carriers. Polysaccharide-based delivery systems also have some additional advantages including availability at large scale, relatively low cost, low toxicity and immunogenicity, high biocompatibility, and biodegradability [55,59]. Consequently, the polysaccharide-based, microbiota-triggered system is promising strategy for colon-specific drug delivery. However, polysaccharides-based delivery systems also have some potential drawbacks, which include broad range of molecular weights and variable chemistry of polysaccharides [59,60]. In addition, low solubility in most organic solvents limits the chemical modification of polysaccharides, while hydrophilicity and excessive aqueous solubility of polysaccharides may cause the early and undesirable drug release in the upper GI tract [60,61]. Accordingly, cross-linking agents are often used to overcome this issue. In addition, the lack of film forming ability, along with swelling and solubility characteristics of polysaccharides limits their application for colonic drug delivery.

To overcome these issues and also to avoid premature drug release in the upper GI tract, polysaccharide-based systems can be prepared by using the combination of polysaccharides and polymers. For example, water insoluble polymers such as Eudragit RS and ethyl cellulose are commonly used along with various polysaccharides for colonic drug delivery [62]. Overall, the use of blended mixture of polysaccharides or other polymers appeared to be more effective in achieving colon-specific drug delivery compared to the use of a single polysaccharide [62]. The drug release rate is dependent on the nature and the concentration of polysaccharides in the combined mixture. Recently, Song et al. [63] developed an oral drug delivery system with programmed drug release and magnetic resonance imaging properties for orthotopic colon cancer therapy. They selected polyacrylic acid (PAA) as a pH-responsive polymer and chitosan (CS) as an enzyme sensitive moiety degradable by β-glycosidase in the colon, which were anchored on Gd^3+^-doped mesoporous hydroxyapatite nanoparticles (Gd-MHAp-NPs). After oral administration, CS and PAA could prevent premature drug release and enhanced drug concentrations at the colon tumor sites [63]. Furthermore, encapsulating both 5-fluorouracil and gefitinib in Gd-MHAp NPs produced a synergistic therapeutic effect, suggesting that this novel delivery system could be a promising treatment strategy for orthotopic colon cancer with programed drug release within the colonic environment [63]. Some of the selected examples for polysaccharide-based systems using the combination of polysaccharides and polymers were presented in Table 1.

Collectively, despite the main drawbacks and limitations of polysaccharide-based delivery systems, their positive aspects and benefits have polysaccharides still used extensively in pharmaceutical applications with various efforts to overcome the barriers.

#### 2.2.2. Phloral^®^ Technology

**Figure 2:**
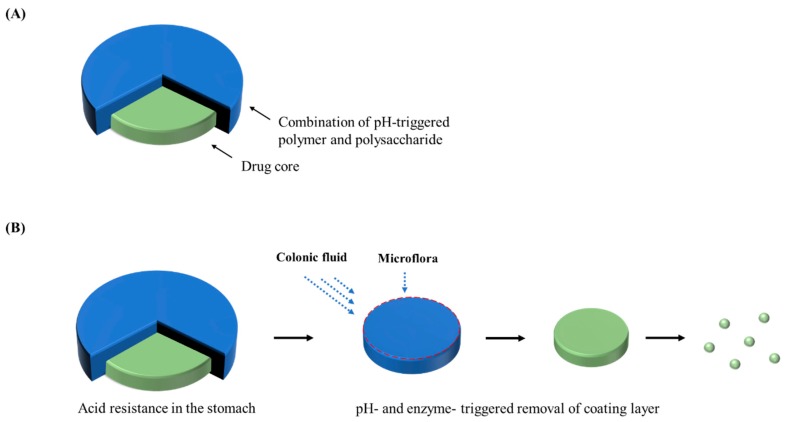
Schematic illustration of Phloral^®^ tablet (**A**) and the drug release from Phloral^®^ tablet (**B**).

Ibekwe et al. [20] reported a novel colonic coating technology which integrated pH-dependent and bacterially-triggered systems into a single layer matrix film. Tablets were film-coated by using a mixture of Eudragit S and biodegradable polysaccharide. Gamma scintigraphy study in human volunteers confirmed the consistent disintegration of these tablets in the colon regardless of feeding status, suggesting that this dual-mechanism coating may overcome the limitation of single trigger systems and improve the colonic drug targeting [20]. Subsequently, Phloral^®^ (Figure 2) coating technology demonstrated the precise and fail-safe drug release in the colon in both healthy and diseased states [81]. This system consists of an enzyme-sensitive component (natural polysaccharide) and a pH-dependent polymer, where these pH and enzymatic triggers work in a complementary manner to facilitate site-specific release [81]. Even if the dissolution threshold of the pH-dependent polymer is not reached, the enzyme-sensitive component is independently digested by enzymes secreted by colonic microflora. This additional fail-safe mechanism overcomes the limitations of conventional pH-dependent systems. This innovative technology has been validated in clinical studies for consistent drug release with reduced-intra subject variability in patients and healthy subjects [81,82]. It is also applicable for the oral delivery of macromolecules such as peptides, proteins, and vaccines. Recently, Dodoo et al. [83] investigated the applicability of this technology in the colonic delivery of probiotics. The commercial products as well as in-house freeze-dried *Lactobacillus acidophilus* strain were encapsulated into capsules using dual-trigger coating technology to target the delivery into lower small intestines or colon. The viabilities of approximately 90% were retained after these capsules were exposed to gastric environment for 2 h while the unencapsulated probiotics showed poor tolerance to the gastric environment [83]. Based on a comparative cohort analysis in patients, Allegretti et al. [84] also demonstrated the effective colon-targeting of the fecal microbiota transplantation capsules coated with a blend of enzyme-triggered and pH-responsive polymers.

Opticore™ that stands for optimized colonic release is a novel starch-based coating technology. It has been developed based on the Phloral^®^ technology and utilizes both pH-triggered and enzymatic-triggered release. This coating technology consists of two trigger systems in an outer coating layer and an accelerator in an inner coating layer to ensure the consistent drug release within the colon [82].

### 2.3. Ligand/Receptor-Mediated Drug Delivery System

**Figure 3:**
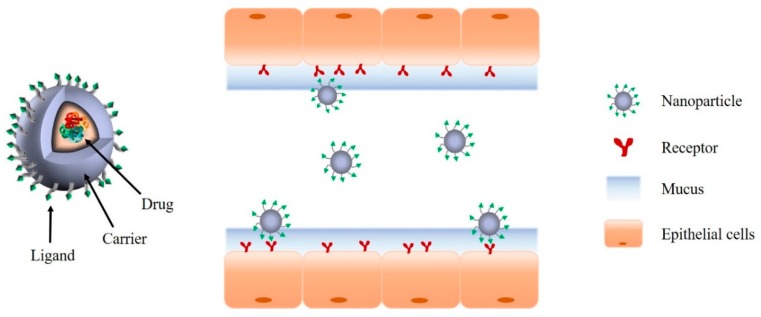
Schematic illustration of representative ligand/receptor-mediated drug delivery system.

For a more effective local treatment of colonic disease with reduced toxic side effects, ligand/receptor-mediated systems have been explored that increase target specificity via the interaction between targeting ligands on the carrier surface and specific receptors expressed at disease sites (Figure 3) [85]. Ligand/receptor-mediated system can be designed using various ligands (e.g., antibodies, peptides, folic acid, and hyaluronic acids) selected based on the functional expression profiles of specific receptors/proteins at the target cells/organs. It can be also combined with pH-dependent systems to maximize its GI stability and site specificity, if needed. Some of the ligands used in colon specific delivery are as described below.

#### 2.3.1. Antibodies

Harel et al. [86] prepared anti-transferrin receptor antibody-conjugated liposomes, demonstrating better cellular internalization of the conjugated liposomes than unconjugated liposomes. Furthermore, anti-transferrin receptor antibody-conjugated liposomes exhibited preferential distribution to the inflamed mucosa rather than normal mucosa, resulting in greater accumulation at the site of inflammation (more than 4-fold higher) when compared to that of normal mucosa. Xiao et al. [87] also developed nanoparticles fabricated with single-chain CD98 antibodies on their surface (scCD98-functionalized) for IBD therapy. CD98 is a heterodimeric neutral amino acid transporter, which is overexpressed in intestinal macrophages and colonic epithelial cells in mice with colitis. scCD98-functionalized nanoparticles exhibited a high affinity for CD98-overexpressed cells [87]. In mice with colitis, scCD98-functionalized nanoparticles containing CD98 siRNA (siCD98) reduced the expression levels of CD98 and the severity of colitis in mice.

#### 2.3.2. Folic Acid

Folic acid, a water-soluble vitamin, is a tumor-selective targeting ligand because the folate receptor is overexpressed in many types of cancers [88]. Many studies have demonstrated that nanoparticles decorated with folic acid can facilitate tumor-selective drug uptake. For example, Xiong et al. [89] reported that folic acid-conjugated liposomes improved the anti-cancer activity of daunorubicin by facilitating folate receptor-mediated drug uptake. Handali et al. [90] also fabricated folic acid (FA)-conjugated liposomes containing 5-fluorouracil (5-FU). 5-FU loaded FA-liposomes exhibited higher cytotoxicity and significantly reduced tumor volume when compared to free drug. These results indicate that folic acid-targeted liposomes may be an effective drug carrier that can increase selective drug delivery to cancer cells. Previously, Zhang et al. [42] had also investigated a folate-modified self-microemulsifying drug delivery system (FSMEDDS) containing curcumin as a means of improving drug solubility as well as its delivery to the colon. Their results confirmed that an FSMEDDS could reach the colon efficiently and release its drug payload rapidly [42]. Furthermore, the FSMEDDS formulation could actively target tumor cells overexpressing folate receptors, indicating that an FSMEDDS may be a promising carrier for the colonic delivery of curcumin.

#### 2.3.3. Hyaluronic Acid

Hyaluronic acid (HA) is a natural polysaccharide consisting of disaccharide units of d-glucuronic acid and *N*-acetyl-d-glucosamine. Since HA has a high affinity for the CD44 receptor, which is overexpressed in various cancers, HA-conjugated drug delivery systems have been examined for target-selective drug delivery [91]. For example, previous studies [91,92] have examined the effectiveness of HA-modified mesoporous silica nanoparticles targeting the CD44-overexpressing cancer cells. Vafaei et al. [93] developed self-assembled HA nanoparticles as colonic carriers of budesonide for targeting inflamed intestinal mucosa. Budesonide loaded HA nanoparticles exhibited higher uptake in inflamed cells over-expressing CD44 receptors, leading to a decrease in IL-8 and TNF-α secretion in an inflamed cell model [93]. Accordingly, HA-conjugated nanoparticles appear to be a promising targeted drug delivery system for IBD treatment.

Xiao et al. [94] investigated an HA nanoparticle-based combination chemotherapy to create synergistic, targeted drug delivery system for colon cancer therapy. They prepared HA-functionalized camptothecin (CPT)/curcumin (CUR)-loaded polymeric NPs (HA-CPT/CUR-NPs) approximately 289 nm in size with a negative zeta potential. HA-CPT/CUR-NPs exhibited significant cancer-targeting capability against Colon-26 cells [94]. They also investigated a simultaneous delivery system of curcumin (CUR) and CD98 siRNA (siCD98), using hyaluronic acid (HA)-functionalized polymeric nanoparticles [95]. Compared to the single drug-based monotherapy, co-delivery of siCD98 and CUR by HA-functionalized nanoparticles exhibited an enhanced therapeutic effect against ulcerative colitis by protecting the mucosal layer and alleviating inflammation [95]. Therefore, HA-functionalized polymeric nanoparticles may be an efficient colonic delivery carrier for combination drug therapy. Recently, Prajapati et al. [96] developed HA-conjugated PEGylated multi-walled carbon nanotubes containing gemcitabine (GEM/HA-PEG-MWCNTs) for colon cancer targeting. HA was conjugated to the surface of PEGylated multi-walled carbon nanotubes (MWCNTs). This formulation showed promising results for effective colon cancer targeting including improved anti-proliferative activity and pharmacokinetic behaviors [96].

#### 2.3.4. Peptides

Peptide gains a great attention as a potential ligand for targeted drug delivery. Peptides possess many advantages including biocompatibility, cost-effectiveness, chemical diversity, and stimuli responsiveness [97,98]. In addition, compared to small molecule ligands, peptide ligands exhibit much higher binding affinity and specificity due to the large binding interfaces with receptors [99,100]. Peptide ligands are also advantageous due to their accessibility of high-throughput screening and ease of synthesis by using automated solid-phase peptide synthesis devices. Furthermore, the metabolic instability by proteases can be overcome via the modification of the peptide sequences, promoting the application of peptide ligands in targeted drug delivery systems. Particularly, peptide-conjugated drug delivery systems are explored as a viable approach for tumor-targeted drug delivery. For example, Ren et al. [101] investigated the application of synthesized 12-residue peptide (TWYKIAFQRNRK, TK peptide) for the colon-specific delivery of anticancer drugs. TK has high affinity to integrin α_6_β_1_, subtype of integrins that is upregulated in human colon cancer cells. Therefore, TK peptide was conjugated to doxorubicin-loaded PEG-PLA micelles as a targeting ligand. This TK-conjugated micelles exhibited significantly stronger cytotoxicity and more effectively penetrated the tumor spheroids, suggesting TK peptide as a promising targeting ligand for colon-targeted therapy [101]. Guo et al. [102] fabricated colon-specific nanoparticles co-modified with amphipathic chitosan derivatives (ACS) and cell penetration peptide (CPP) to improve the oral bioavailability of insulin. ACS modification could protect CPPs from degradation in the upper GI tract and achieved colon-specific drug delivery. Once CS-CPP NPs reached the colon, ACSs on the surface of the NPs were gradually degraded and the exposed CPPs facilitated the drug penetration across the colonic epithelium [102]. The results from in vitro and in vivo evaluation suggest that CS-CPP NPs may be an effective colon-specific drug delivery system to improve the oral absorption of proteins and peptides.

### 2.4. Magnetically-Driven Drug Delivery System

**Figure 4:**
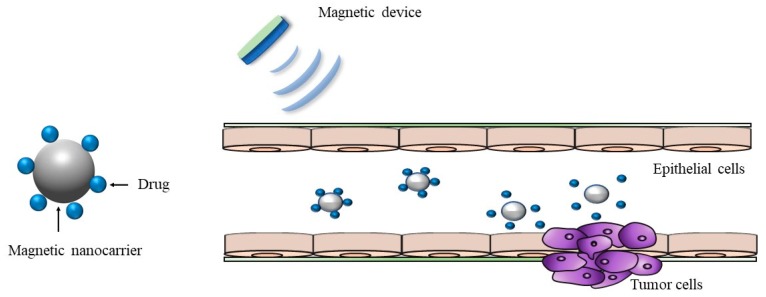
Schematic illustration of magnetic nanocarrier drug delivery system.

Magnetic microcarriers including magnetic microspheres, magnetic nanoparticles, magnetic liposomes, and magnetic emulsions are emerging novel formulations for controlled and targeted drug delivery (Figure 4). To improve the targeted treatment of colorectal cancer by mAb198.3 (a FAT1-specific monoclonal antibody), Grifantini et al. [103] developed two different novel drug delivery systems having magnetic properties to improve the targeted treatment of colorectal cancer by mAb198.3 (a FAT1-specific monoclonal antibody), where mAb198.3 was directly bound to super-paramagnetic nanoparticles or embedded into human erythrocyte-based magnetized carriers. They observed that both systems were very effective at targeting colon cancer cells and inhibiting cancer growth at significantly lower antibody doses [103]. This study demonstrated the potential of magnetically-driven drug delivery systems at improving the bioavailability and target specificity of anti-FAT mAb198.3, opening a new avenue for colon-targeted drug delivery [103]. Another previous study improved the efficacy of hydrocortisone using a magnetic belt on rats [104]. This nanodevice consisted of magnetic mesoporous silica microparticles loaded with hydrocortisone. The outer surface of the drug-loaded nanoparticles was functionalized with a bulky azo derivative with urea moieties. The nanodevices remained capped at neutral pHs, but a noticeable payload release occurred in the presence of sodium dithionite because it reduced the azo bonds in the capping joint [104]. They also observed the improved efficacy in rats wearing magnetic belts, particularly being more effective when a magnetic field was externally applied to lengthen the retention time in the areas of interest [104]. This study demonstrated that the use of a magnetic belt increased the drug efficacy in the treatment of IBD due to enhanced retention time of the drugs in the colon. Recently, Kono et al. [105] developed magnetically-directed cell delivery systems via the incorporation of superparamagnetic iron oxide nanoparticles (SPIONs) and plasmid DNA (pDNA) into RAW264 murine macrophage-like cells. They also demonstrated that this magnetic cell delivery system could enhance the colonic delivery of macrophages in mice [105].

## 3. Complementary Tools for Designing the Effective Colonic Drug Delivery Systems

Optimizing drug formulations using traditional approach requires many experiments including various in vitro and in vivo tests, which are often tedious, time-consuming, high-cost tasks [106]. Furthermore, many drug delivery systems are promising in vitro but often fail in vivo, which is mainly due to the lack of mechanistic insight from experiments based on trial and error [107]. The computational methods including molecular modeling and simulation, data mining, and an artificial intelligence technique are useful to expedite the rational formulation design. It can save much experimentation effort and time by identifying the critical factors for the optimization of formulations and selecting the promising candidates for further experimental confirmation. For example, Metwally and Hathout [106] have proven that the combined use of several chemo/bio informatics and statistical tools could effectively predict the loading efficiency of drugs in a carrier and also elucidate the effect of certain molecular descriptors of drugs on their docked binding energies on carriers [106]. This would allow the accurate estimation of entrapment efficiencies and loading capacity in drug delivery systems without exhaustive laboratory experiments.

In general, computer modeling techniques allow the identification of critical variables for the optimization of formulations and also provide detailed information on molecular interaction of drug-carrier, entrapment efficiency, drug distribution/localization in delivery systems, stability, drug release behavior, and so on [107]. Consequently, these computational methods are capable of complementing experiments and assist more rational formulation design and optimization. Furthermore, integration of such computational tools with other technology for targeted drug delivery has led to a new era of revolutionized drug delivery systems such as electronic drug delivery devices and radiofrequency drug delivery devices. Some of selected examples on the application of computational and device-based approaches to assist the colon-specific formulation design are discussed below.

### 3.1. Computer-Assisted Formulation Design

Chemo/bio-informatics tools and statistical methods are useful to assist the rational formulation design and complement experiments. The computational approach is also applicable for investigating the performance of drug delivery systems along with the effect of various environmental conditions including pH, temperature, salt concentration, external stimulus, and the interaction with other biomolecules in the body. Patra et al. [108] synthesized biopolymeric glycogen-based fluorescent gel for the colon specific drug delivery of metronidazole and ciprofloxacin. In addition to the experimental evaluation, they carried out ab initio molecular dynamics study to investigate the probable interaction of drugs with hydrogel in molecular level. They also performed Quantum mechanical/Molecular mechanics calculations to investigate the pH-responsive swelling and drug release from the developed hydrogel [108]. The results indicated the physical interaction between hydrogel and drug molecules during its swelling and also confirmed the pH-dependent drug release patterns, in correspondence with the experimental observations [106]. Markovic et al. [109] suggest a novel phospholipid (PL)-based prodrug approach for colon-specific drug delivery, by targeting the phospholipase A_2_ (PLA_2_) as the PL-prodrug activating enzyme overexpressed in the inflamed colonic tissues. First, they selected Fmoc (fluorenylmethyloxycarbonyl) as a model compound and synthesized PL-Fmoc conjugates with different linker lengths between the PL and the drug moiety. Then, they evaluated experimentally the PLA_2_-mediated activation of the PL-Fmoc conjugates. Furthermore, they also conducted a novel molecular dynamics simulation of the transition state of the conjugate in the PLA_2_ enzyme complex, in order to determine the optimal linker length for the clinically relevant drug in ulcerative colitis such as methotrexate. The simulation results indicated that the free energy of the PL-prodrug binding to the transition state geometry of the enzyme dictated the rate of PLA_2_-mediated activation, and the linker length of 6 should be optimal with the highest extent of PLA_2_-mediated activation, while shorter linkers were activated to a lower extent. This study suggests that these highly reliable computational methods allow the optimization of the chemical structure of the molecular linker between the PL and drug moiety and also reduce the amount of chemical synthesis needed for the development of effective prodrugs for colon-specific delivery [109].

Recently, SIMGI (SIMulator Gastro-Intestinal) as an automated in silico model, enables to simulate the physiological processes in GI tract and also to reproduce the microbiota in the colon [110]. This computational model is applicable to examine the food effects on modulating the gut microbiota and its metabolic activity. This model may be beneficial while designing a more efficient colon-targeted delivery system in a quick and economical way, although it has a short history, and some drawbacks of this system are on the way to be overcome [110].

Collectively, computational approach can offer an efficient toolbox for designing the optimal colon-targeted drug delivery systems and predicting the in vivo performance of the developed formulations.

### 3.2. Electronic Device-Assisted Formulation Design

For the successful development of colon-specific drug delivery systems, in vivo characterization of drug absorption throughout the GI tract is essential. Accordingly, there is a strong need for a quick and simple way to precisely and reliably assess the drug release properties within the GI tract to determine whether the tested formulation is valid for modified drug release. In that sense, the use of electronics brings a new approach for integration of data from multiple sources. IntelliCap^®^ is the world’s first intelligent electronic drug delivery and monitoring device, which combines controlled drug release, patient monitoring, and real-time wireless communication [111,112]. Since this electronic capsule features real-time wireless data recording, it can provide care givers the ability to monitor the progress of the capsule through the GI tract. Furthermore, simultaneous measurement of pH and transit, along with accurately targeting drug delivery, makes in vivo data available for a formulation design [111,112]. Consequently, IntelliCap^®^ technology provides a fast and convenient tool for the controlled drug release to specific sites in the GI tract. By using Intellicap^®^ system, Maurer et al. [50,51] confirmed the ileo-colonic drug release of ColoPulse tablets in humans, supporting that the ColoPulse system is a promising colonic drug delivery system.

In addition to many benefits, there are also some disadvantages associated with electronic capsules including high cost, manufacturing difficulties, biocompatibility issues, and potential risk of device failure [111,112]. Hence, there should be continuous efforts to overcome these disadvantages in order to make electronic delivery systems more widely compatible. In the long run, electronic drug delivery system is a promising new approach for the controlled drug release at the desired target sites.

## 4. Summary

Colon-targeted drug delivery is an essential strategy for more effective local treatment of colonic diseases such as IBD and colorectal cancers. It may offer many benefits over conventional dosage forms in terms of safety, efficacy, and patient compliance. In addition, colon-targeted delivery systems are applicable to improve the systemic exposure of acid-and/or enzyme-labile drugs including macromolecules. Although advancements in biotechnology and protein engineering have expanded the therapeutic application of proteins and peptides, most biologics on the pharmaceutical market are in parenteral formulations due to their low permeability and physicochemical and metabolic instability in the GI tract. Therefore, colon-targeted delivery systems gain great attention as an effective formulation strategy to improve the oral bioavailability of macromolecules.

In this review, various formulation approaches to develop the effective colon-targeted delivery systems were discussed with some case studies. All of these formulation strategies possess their own advantages and disadvantages, requiring continuous refinement to improve their therapeutic efficiency. For the successful development of colon-targeted drug delivery systems, it is imperative to consider the physiological and pathophysiological properties of the colon and the microenvironment surrounding disease site(s). However, the dynamic changes in the physiological conditions in GI tract and also the pathophysiological changes in the microenvironment surrounding disease sites make optimal formulation design more complicated, often leading to in vivo failure with lack of site specificity. For example, the dynamic pH change in GI tract by many internal and external factors may attenuate the efficiency of pH-dependent drug release systems, resulting in premature drug release in upper GI tract, or incomplete drug release at the target site. Accordingly, the combined systems of the different release-triggering mechanisms are actively pursued to overcome the pathophysiological variability issues. In addition, nano-/micro-particles hold great potential for enhancing drug targeting as well as drug uptake. Therefore, various formulations with particle size reduction may be beneficial for colon-targeted drug delivery. Computer-assisted and electronic device-assisted formulation design also allow more rational formulation design and optimization, reducing the time and cost for experiments. Taken together, to overcome the limitations of current formulation approaches, there should be continuous efforts to invent new formulation technologies. These efforts include the discovery of the new biocompatible functional materials, the development of more precise drug delivery devices, and utilization of big data.

## Figures and Tables

**Table 1:** Selected examples of polysaccharides-based colonic delivery system.

Polysaccharide	Delivery System	API	Mechanism	Ref
Alginate	Calcium alginate beads coated with Eudragit^®^ S-100	Curcumin	pH responsive, enzyme sensitive, and mucoadhesiveness	[64]
Alginate	Calcium alginate-Carboxymethyl cellulose beads	5-fluorouracil	pH responsive, enzyme sensitive, and mucoadhesiveness	[65]
Alginate/Chitosan	Chitosan succinate-Sodium alginate beads	Capecitabine	pH responsive, enzyme sensitive, and mucoadhesiveness	[66]
Alginate/Portulaca	Portulaca-Sodium alginate/Borax composite microbeads	5-fluorouracil	pH responsive, enzyme sensitive, and mucoadhesiveness	[67]
Alginate/Chitosan/Konjac glucomannan	Chitosan coated konjac glucomannan/Sodium alginate/Graphene oxide microspheres	Ciprofloxacin	pH responsive, enzyme sensitive, and mucoadhesiveness	[68]
Alginate/Pectin	Sodium alginate liposome coated with pectin	Salmon calcitonin	pH responsive, enzyme sensitive, and mucoadhesiveness	[69]
Alginate/Chitosan	Alginate/Chitosan microcapsules	Interleukin-1Ra	pH responsive, enzyme sensitive, and mucoadhesiveness	[70]
Alginate/Chitosan/Kappa- carrageenan	Dual layered pH-sensitive Alginate/Chitosan/Kappa- carrageenan microbeads	5-Flurouracil	pH responsive, enzyme sensitive, and mucoadhesiveness	[71]
Pectin/Chitosan	Pectin/Chitosan beads containing drug loaded in potato starch	Doxorubicin	Enzyme sensitive and mucoadhesiveness	[58]
Pectin/Chitosan	Modified citrus pectinate-chitosan nanoparticle (MCPCNP)	Curcumin	Enzyme sensitive and mucoadhesiveness	[72]
Pectin/Chitosan	Modified citrus pectinate-chitosan nanoparticle (MCPCNP)	Cetuximab Curcumin	Enzyme sensitive and mucoadhesiveness	[73]
Pectin/Chitosan	Chitosan-Zinc-Pectinate-Polyethylene glycol (PEG) nanoparticles (NPs)	Resveratrol	Enzyme sensitive and mucoadhesiveness	[74]
Chitosan/Nutriose	PEG-containing vesicles coated with chitosan/nutriose	Quercetin	Enzyme sensitive and mucoadhesiveness	[75]
Pectin	Pectin-Zinc acetate beads coated with Eudragit S100	Pterostilbene	pH responsive and enzyme sensitive	[76]
Pectin	Pectin/Polyethylene glycol hydrogel system containing in-situ mineralized calcium carbonate microparticle	Bovine serum albumin	Enzyme sensitive and mucoadhesiveness	[77]
Guar Gum	Guar Gum capped mesoporous silica nanoparticles	5-Flurouracil	Enzyme sensitive and Nanoparticle	[78]
Guar Gum	Ethylene glycol dimethacrylate cross-linked guar gum oleate-*graft*-poly (methacrylic acid) hydrogel	Ibuprofen	pH responsive, enzyme sensitive, and mucoadhesiveness	[79]
Inulin	Cinnamate inulin microsphere hydrogel system	Methotrexate	Enzyme sensitive and mucoadhesiveness	[80]
